# Isolation of a Monoclonal Antibody and its Derived Immunosensor for Rapid and Sensitive Detection of 17β-Estradiol

**DOI:** 10.3389/fbioe.2022.818983

**Published:** 2022-03-28

**Authors:** Jingru Liang, Hang Dong, Fei Xu, Baowei Li, Haimei Li, Limei Chen, Mei Li, Yingchu Liu, Guosheng Jiang, Jinhua Dong

**Affiliations:** ^1^ Key Laboratory for Biological Medicine in Shandong Universities, Weifang Key Laboratory for Antibody Medicine, School of Life Science and Technology, Weifang Medical University, Weifang, China; ^2^ School of Clinical Medicine, Peking University, Beijing, China; ^3^ School of Clinical Medicine, Beijing University of Chinese Medicine, Beijing, China; ^4^ College of Basic Medicine, Binzhou Medical University, Yantai, China; ^5^ World Research Hub Initiative, Institute of Innovative Research, Tokyo Institute of Technology, Yokohama, Japan

**Keywords:** estradiol, phage display, monoclonal antibody, immunosensor, rapid detection

## Abstract

Estrogens are effective for stimulating several functions in living organisms and for regulating cancer development by promoting cell proliferation. Estradiol can disrupt the reproductive and endocrine systems, leading to the development of various diseases. In this study, the monoclonal antibody ESC9 was developed by immunizing mice with a 17β-estradiol (E2) conjugate, preparing an antibody phage display library, and screening monoclonal antibodies from the prepared library. An antibody with the same sequence as that of ESC9 has not been reported previously. The equilibrium dissociation constant between ESC9 and E2 was found to be 43.3 nM. Additionally, we generated an ESC9-derived immunosensor named as the ESC9 Quenchbody (Q-body), which can rapidly and sensitively detect E2. The assay can be completed within 2 min with a limit of detection of 3.9 pg/ml and half-maximal effective concentration of 154.0 ng/ml. Serum E2 levels were measured using the ESC9 Q-body without pretreatment with serum and with a high recovery rate of 83.3–126.7%. The Q-body immunosensor shows potential for clinical applications based on its excellent detection speed and sensitivity.

## Introduction

Estrogens effectively stimulate several functions in living organisms and regulate cancer development by promoting cell proliferation (Russo and Russo, 2006; Kumar et al., 2018). As one of the most active steroidal estrogens, 17β-estradiol (E2) has been widely examined. High or low levels of estradiol can disrupt the reproductive and endocrine systems, leading to the development of many diseases. Estradiol levels in the human serum can be used clinically to diagnose endocrine or gynecological disorders and male and female infertility, to assess male and female gonadal function and the post-menopausal status, and as a diagnostic indicator for tumors, such as ovarian and pituitary tumors (Rosenfeld et al., 2001; Schlegel, 2012; Luine, 2014; Leivo et al., 2019).

The main methods used to detect estradiol are chromatography (Asadi Atoi et al., 2019), enzyme-linked immunosorbent assay (ELISA) (Silva et al., 2013), radioimmunoassay (Saumande, 1981), chemiluminescence (Leivo et al., 2019), electrochemiluminescence (Ojeda et al., 2012) and homogeneous enzyme immunoassay (Chiu et al., 2011). However, these assays are complex and some exhibit limited reproducibility, stability, and sensitivity. Among these methods, chromatography is a relatively sensitive assay with high accuracy; however, this assay requires several treatments of the sample in advance, costly instruments, and expert personnel. Therefore, the use of chromatography assays in clinical diagnoses is not preferred.

In contrast, Quenchbody (Q-body), a fluorescent biosensor based on antigen-antibody reactions, is simple to operate, highly specific, and can significantly improve the sensitivity and accuracy of the traditional assay. The Q-body is a recombinant antibody fragment in which the N-terminus is specifically labeled with a fluorescent dye, and the fluorescence is quenched by a tryptophan residue in the variable region of the antibody based on photo-induced electron transfer (Abe et al., 2011; Dong et al., 2020b; Dong and Ueda, 2021). Specific binding of the antigen to the antibody results in quenching of the fluorescent dye inside the antibody. The dye is displaced to the outside of the antibody, where de-quenching occurs, resulting in an antigen-dependent increase in fluorescence intensity (Dong et al., 2020a).

Monoclonal antibodies are the basis of immunoassays, and hybridoma technology (Kohler and Milstein, 1975) and molecular phage display technology (Smith, 1985) are two important methods for developing monoclonal antibodies. Phage display technology can correlate the genotype and phenotype of antibodies and can be used to rapidly develop monoclonal antibodies and evaluate antibody activity. In the present study, an anti-E2 antibody was developed by immunization of mice and the preparation and screening of a phage display antibody library. An E2 Q-body was prepared by labeling the N-terminus of an anti-E2 antibody with a fluorescent dye. A concentration-dependent increase in fluorescence intensity was detected following addition of E2, which was used to accurately determine serum E2 levels. This fluorescent biosensor, which is based on the specific binding of antigens and antibodies, can improve the accuracy of E2 detection and facilitate standardization of E2 test results.

## Materials and Methods

### Materials

The strains *Escherichia coli* TG-1 used to construct the phage display antibody library and *E. coli* DH5α for gene cloning were purchased from Agilent Technologies (Santa Clara, CA, United States). *Escherichia coli* SHuffle T7 Express lysY was purchased from New England Biolabs (Ipswich, MA, United States) (Bessette et al., 1999; Levy et al., 2001). The E2 ovalbumin conjugate (E2-OVA) for immunization and E2 bovine serum albumin conjugate (E2-BSA) for antibody screening were purchased from Wuhan Huamei Biotechnology Co., Ltd (Wuhan, China). BALB/c mice were purchased from Jinan Yuepeng Experimental Animal Breeding Center (Jinan, China). The primers used in this study were synthesized by Shanghai Sangon Biotech Co. Ltd (Shanghai, China). Restriction enzymes were purchased from New England Biolabs. Unless otherwise specified, all reagents were purchased from Aladdin Industrial Corporation (Shanghai, China) or Shanghai Sangon Biotech.

### Mouse Immunization

**FIGURE 1:**
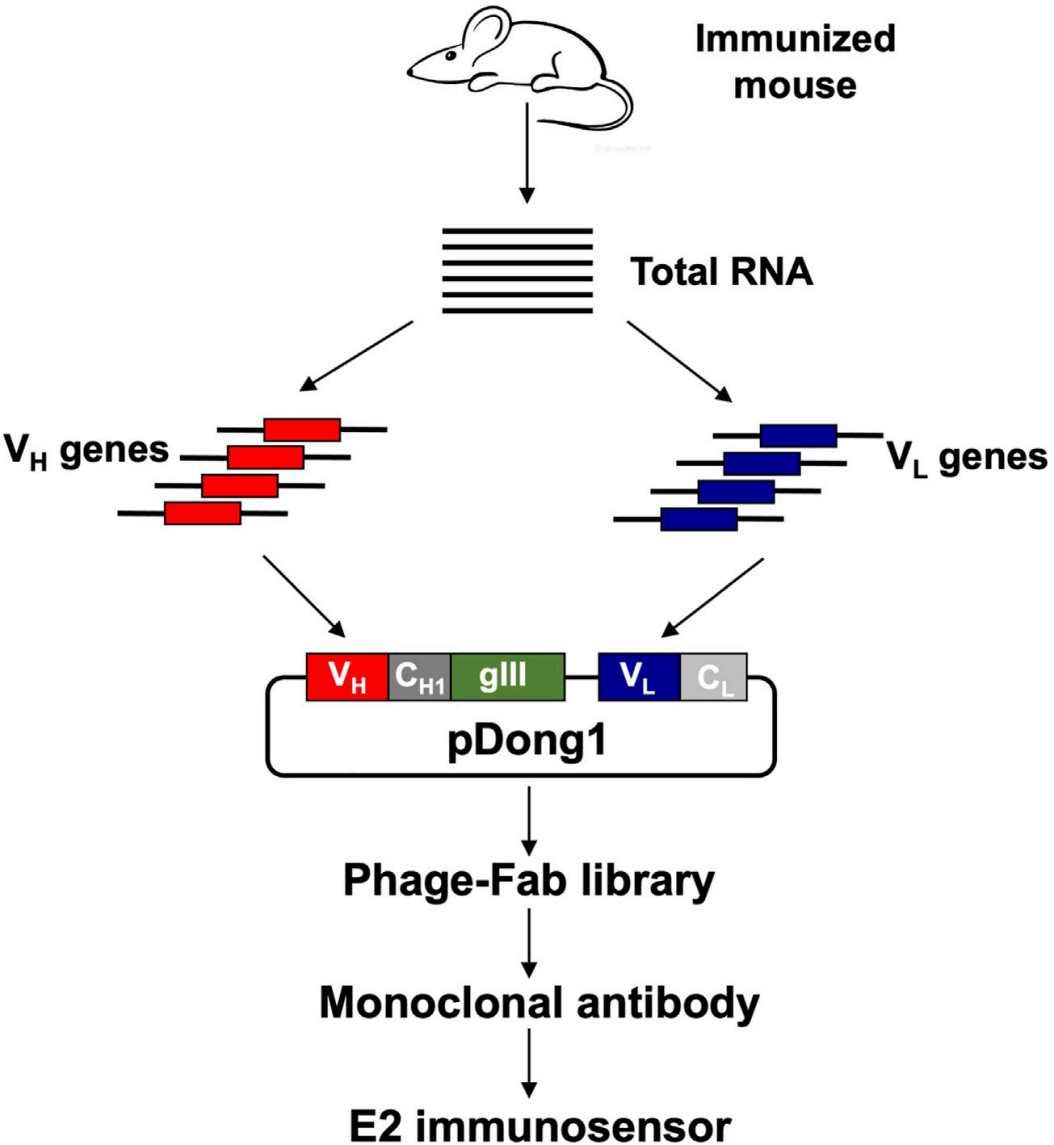
Flow chart of antibody library construction and monoclonal antibody selection. V_H_: variable region of antibody heavy chain; V_L_: variable region of antibody light chain; C_H1_: constant region 1 of heavy chain; C_L_: constant region of light chain.

As shown in Figure 1, an anti-E2 antibody was obtained by immunizing BALB/c mice with the E2-OVA conjugate, preparing an antibody phage display library, and screening for monoclonal antibodies from the library. The developed monoclonal antibody was used to construct a fluorescent immunosensor for detecting E2. First, two BALB/c mice were immunized with 100 μl E2-OVA at a concentration of 100 μg/ml. Three immunizations were performed at 2-week intervals. Blood was collected from the tails of the mice at 1 week after the last antigen injection. ELISA was performed using immobilized E2-BSA to confirm the titer of anti-E2 antibodies in the blood of mice. The experimental protocol was established according to the ethical guidelines of the Declaration of Helsinki and approved by the Animal Ethics Committee of Weifang Medical University.

### Construction of Phage Display Antibody Library

After confirming the presence of anti-E2 antibodies in the blood, the mice were sacrificed, and their spleens were extracted for total RNA extraction. TRIzol (Life Technologies, Carlsbad, CA, United States) was used for total RNA extraction according to the manufacturer’s instructions. Reverse transcription-polymerase chain reaction (RT-PCR) was performed with 1 µl of total RNA to amplify genes in the antibody heavy chain variable region (V_H_) and light chain variable region (V_L_) using a PrimeScript One Step RT-PCR kit from Takara Bio (Shiga, Japan) according to the manufacturer’s instructions. The murine antibody-specific primers used for this step are listed in Supplementary Table S1. The V_H_ and V_L_ genes were treated with the restriction enzymes *Sfi*I/*Xho*I and *Sal*I/*Not*I, respectively, and cloned into the phage display vector pDong1. The recombinant plasmids were used to transform *E. coli* TG-1 to prepare a phage display antibody library according to previously reported protocols (Dong et al., 2009).

### Screening of Monoclonal Antibody

Sodium bicarbonate solution (100 µl) containing 10 μg/ml of E2-BSA was added to a 96-well microplate, which was then incubated at 4°C overnight. The next day, the cells were washed with PBS containing 0.1% Tween 20 (PBST), after which 200 μl phosphate-buffered saline (PBS) containing 2% skim milk powder (MPBS) was added to each well and incubated for 2 h to block any non-specific binding. After incubation and washing, 100 μl of phage solution containing 1 × 10^10^ colony forming units of phage displaying the antibody library was added to each well. After shaking for 1 h and incubating for another 1 h, the phage antibody bound to E2 was eluted with 1 mg/ml trypsin after washing. The eluted phage antibody was used to infect TG-1, and the phage display antibody library R1 was prepared at 30°C overnight. The above steps were repeated for the second and third rounds of selection and preparation of phage display antibody libraries R2 and R3, respectively. The microplates were coated with E2-BSA, and ELISA was performed to compare the E2-binding activity of the antibody libraries, including the primary phage library (R0), R1, R2, and R3. *Escherichia coli* TG-1 was infected with the phage library that exhibited the highest E2-binding activity and plated on Luria-Bertani (LB) solid medium containing 100 μg/ml ampicillin. After overnight culture at 37°C, 96 colonies were selected to prepare phage-display antibodies. Phage ELISA was performed to select positive clones, which were further cultured for plasmid extraction and sequence analysis.

### Vector Construction for Expression of Antigen-Binding Fragment (Fab)

The flowchart for constructing the vector expressing ESC9 Fab is shown in Supplementary Figure S1. The V_H-_C_H_1 and V_L_ genes of the selected positive clone ESC9 were amplified from pDong1-ESC9 using the primer pairs InfuAgeIESC9Vhback/OverlapEcoRvMycFor and OverlapEcoRvESC9VLback/InfuHindIIIESC9VL, respectively, followed by linking of the V_H_-C_H_1 and V_L_ genes to obtain V_H_-C_H_1-V_L_ by overlap PCR with the primers InfuAgeIESC9Vhback and InfuHindIIIESC9VL. The purified V_H_-C_H_1-V_L_ DNA fragments were then ligated and cloned into the pUQ1H (KTM219) vector (Abe et al., 2014) after double digestion with *Age*I and *Hin*dIII. The positive clone pUQ1H-ESC9 selected for the expression of ESC9 Fab was screened using colony PCR with T7 promoter and T7 terminator primers, followed by sequence analysis. The primers used to construct the Fab expression vectors are listed in Table 1.

**TABLE 1:** Primers for preparation of Fab expression vector.

Primer name	Sequence (5′-3′)	Length (bp)
InfuAgeIESC9VHback	ACT​GCT​CTA​ATG​AGA​CCG​GTG​AGG​TTC​AGC​TGC​AGC​AGT​CT	41
OverlapEcoRvMycFor	GTG​ATA​TCT​CCT​TCT​Aga​TTA​TTA​TGC​GGC​CCC​ATT​CAG​AT	42
OverlapEcoRvESC9VLback	TAG​AAG​GAG​ATA​TCA​CAT​GGA​TAT​TGT​GAT​GAC​GCA​GGC​T	40
InfuHindIIIESC9VLfor	ACG​TTT​GAT​TTC​aAG​CTT​GGT​CCC​AG	26
T7 promoter	TAA​TAC​GAC​TCA​CTA​TAG​GG	20
T7 terminator	GCT​AGT​TAT​TGC​TCA​GCG​G	19

### Expression and Purification of ESC9 Fab

The successfully constructed vector was transformed into SHuffle T7 Express lysY *E. coli* cells, after which 40 µl of the transformed bacteria was spread evenly on LB solid medium containing 100 μg/ml ampicillin and incubated at 37°C overnight. Single colonies were picked and inoculated into 4 ml LB liquid medium containing 100 μg/ml ampicillin and incubated overnight at 250 rpm and 37°C. The overnight culture was transferred to 300 ml of fresh LB broth, to which 0.5 mM isopropyl β-D-thiogalactoside was added. The mixture was incubated at 220 rpm at 16°C for 20 h to induce protein expression.

The supernatant was removed by centrifugation at 8,000 × *g* for 10 min at 4°C. Thereafter, 10 ml of binding/washing buffer (50 mM phosphate, 0.3 M NaCl, 5 mM imidazole, pH 7.4) was added to resuspend the bacterial precipitate. The cells were sonicated on ice and centrifuged at 8,000 × *g* for 10 min at 4°C. The collected supernatant (10 ml) was added to an immobilized metal affinity chromatography resin, spin-bound for 1 h at 4°C, and passed through the column. After equilibrating the column with binding/washing buffer, the column was washed five times to remove heteroproteins. The target proteins were eluted by incubation with elution buffer (50 mM phosphate, 0.3 M NaCl, 500 mM imidazole, pH 7.0) for 1 min. The target protein bands were verified using sodium dodecyl sulfate-polyacrylamide gel electrophoresis (SDS-PAGE).

### ELISA

BSA and E2-BSA (10 μg/ml each) were immobilized in 96-well clear polystyrene microplates and incubated overnight at 4°C. Blocking was performed with MPBS (200 μl) for 2 h, and the plates were washed three times with PBST. Thereafter, 100 μl of the anti-E2 antibody (10 μg/ml) was added, and the mixture was incubated for 1 h at 25°C, followed by three washes with PBST. Anti-6×His tag mouse antibody (100 µl) diluted by 1:2,000 was added, and the plates were incubated at 25°C for 1 h. After three washes with PBST, 100 μl of the rabbit anti-mouse antibody coupled with horseradish peroxidase (HRP) diluted by 1:5,000 was added to the plates and incubated at 25°C for 1 h. After three washes with PBST, 100 μl of color-developing solution was added. The chromogenic solution was composed of 200 μg/ml 3,3,5,5-tetramethylbenzidine and 0.3 μl/ml hydrogen peroxide in 100 mM sodium acetate (pH 6.0). The reaction was terminated by adding 50 μl 10% H_2_SO_4_ to each well after 5 min of color development. Absorbance was measured at 450 nm using an iMark™ microplate reader (Bio-Rad, Hercules, CA, United States), with 630 nm as a reference.

### Kinetic Parameters of ESC9 Fab Binding to E2

E2-BSA was labeled with a biotinylation kit (BBI Life Sciences, Shanghai, China) according to the manufacturer’s instructions to obtain E2-BSA-biotin, and the streptavidin-binding activity of E2-BSA-biotin was verified by performing the following assay: E2-BSA-biotin was coated onto a 96-well plate at 10 μg/ml and incubated at 4°C overnight. After washing and blocking the wells with PBST and MPBS, respectively, streptavidin-HRP (0.2 μg/ml) was added to each well and the plate was incubated at 25°C for 1 h. After washing with PBST, the assay was carried out based on the protocol for ELISA.

E2-BSA-biotin was fixed on a streptavidin (SA) probe, and kinetic parameters were measured using an Octet Red 96e system (FortéBio Corp., Fremont, CA, United States) based on bio-layer interference (BLI) technology. Antibody solutions with different concentrations (0, 50, 100, 200, 300, and 500 nM) were used to detect the binding rate in kinetic buffer (PBS containing 0.02% Tween 20 and 0.1% BSA). The dissociation rates of antibodies were determined. The SA probe was immersed in kinetic buffer without antibody as a negative control. The detection curve and equilibrium dissociation constant (*K*_D_) were obtained using Data Analysis 12 HD (FortéBio).

### Competitive ELISA

E2-BSA (100 µl; 10 μg/ml) was added to the wells of a microplate and incubated overnight at 4°C. The E2-BSA solution was discarded, and the microplate was blocked with 200 μl of MPBS. After washing three times with PBST, 100 µl of ESC9 Fab solution at 10 μg/ml with E2, testosterone, dehydroepiandrosterone, pregnenolone acetate, cortisol (Shanghai Macklin Biochemical Co., Ltd.), and diethylstilbestrol (Macklin) at concentrations of 0, 0.4, 2.0, 10, 50, 250, 1,000, 2000, and 20,000 ng/ml was added to the wells and incubated at 25°C. for 2 h. After washing with PBST three times, HRP-conjugated anti-His mouse antibody diluted by 1:5,000 was added and incubated for 1 h at 25°C. After washing, the assay was performed as previously described in ELISA section. The half-maximal inhibitory concentration (IC_50_) of each competitive assay and cross-reactivity of ESC9 against E2, testosterone, dehydroepiandrosterone, pregnenolone acetate, cortisol, and diethylstilbestrol were calculated.

### Preparation of E2 Q-Body

Fab solution (300 µl; 20 μM) was added to 0.5 mM tris (2-carboxyethyl) phosphine hydrochloride, and the exposed thiol groups were lightly reduced by mixing at 4°C for 20 min in the dark. Next, 2 mM 4-azidobenzoic acid was added, and the mixture was incubated on ice for 10 min. ATTO520-C2-maleimide (ATTO-TEC GmbH, Siegen, Germany) at a final concentration of 200 μM was added to the reaction solution, and the mixture was incubated at 25°C for 2 h. The protein was further purified by adding 60 μl of DYKDDDDK tag antibody-coated agarose beads (Thermo Fisher Scientific, Waltham, MA, United States) and incubating the sample for 2 h at 4°C in the dark with stirring. Free fluorescent dye was removed by washing with PBST at 4°C, followed by centrifugation 5 times at 5,000 × *g* for 10 s each. The Q-body was obtained by adding 100 μl of 3×FLAG peptide at a concentration of 150 μg/ml and incubating the sample for 1 h at 4°C in the dark, followed by centrifugation for 1 min at 5,000 × g. SDS-PAGE was performed to verify the fluorescence modification and purity of the purified Q-body.

### Denaturation Tests of ESC9 Q-Body and Detection of E2

The initial quenching of the Q-body was analyzed by comparing the fluorescence spectra obtained using PBST and PBST containing equal volumes of 7 M guanidine hydrochloride and 100 mM dithiothreitol (GdnHCl/DTT), using a Hitachi F4600 fluorescence spectrophotometer (Tokyo, Japan). The excitation and emission wavelengths of the ATTO520 dye were 520 and 545 nm, respectively. The fluorescence spectra of the ESC9 Q-body were again measured after adding E2 at final concentrations of 1.0, 5.0, 10.0, and 100 pg/ml; 1.0, 10.0, 100.0, and 500.0 ng/ml; and 1.0, 5.0, and 10.0 μg/ml followed by incubation at 25°C for 2 min. The fluorescence intensity of the Q-body at 545 nm in the presence of different concentrations of E2 was normalized by dividing the intensity by that of the Q-body without E2. Normalized values were used to draw a concentration-dependent curve for E2 detection. As shown in Eq. 1, a four-parameter logistic equation was used to fit the concentration-dependent relationship curve, and half-maximal effective concentration (EC_50_) was evaluated using GraphPad Prism eight software (GraphPad, Inc., San Diego, CA, United States). The limit of detection of the assay for E2 was estimated as the antigen concentration equal to the mean blank value plus three standard deviations.

$$y\;=\;d+\frac{a-d}{1+{\left(\frac{x}{c}\right)}^{b}}$$ (1)

### Detection of E2 in Spiked Serum Samples

To evaluate the applicability of the ESC9 Q-body in practical assays, the E2 content in 25% serum was assayed using ESC9 Q-body. Three concentrations of E2 standards (100.0 pg/ml, 1.0 ng/ml, and 100.0 ng/ml) were added to human serum diluted to 25% with PBST and mixed with the ESC9 Q-bodies. The fluorescence intensity of prepared samples was measured at excitation and emission wavelengths of 520 and 545 nm, respectively, from which the fluorescence intensity of diluted serum was subtracted. Finally, the normalized fluorescence intensity of the Q-body probe in the serum samples was interpolated in the concentration-dependent standard curve and the E2 concentrations was calculated.

## Results

### Screening of Monoclonal Antibodies

**FIGURE 2:**
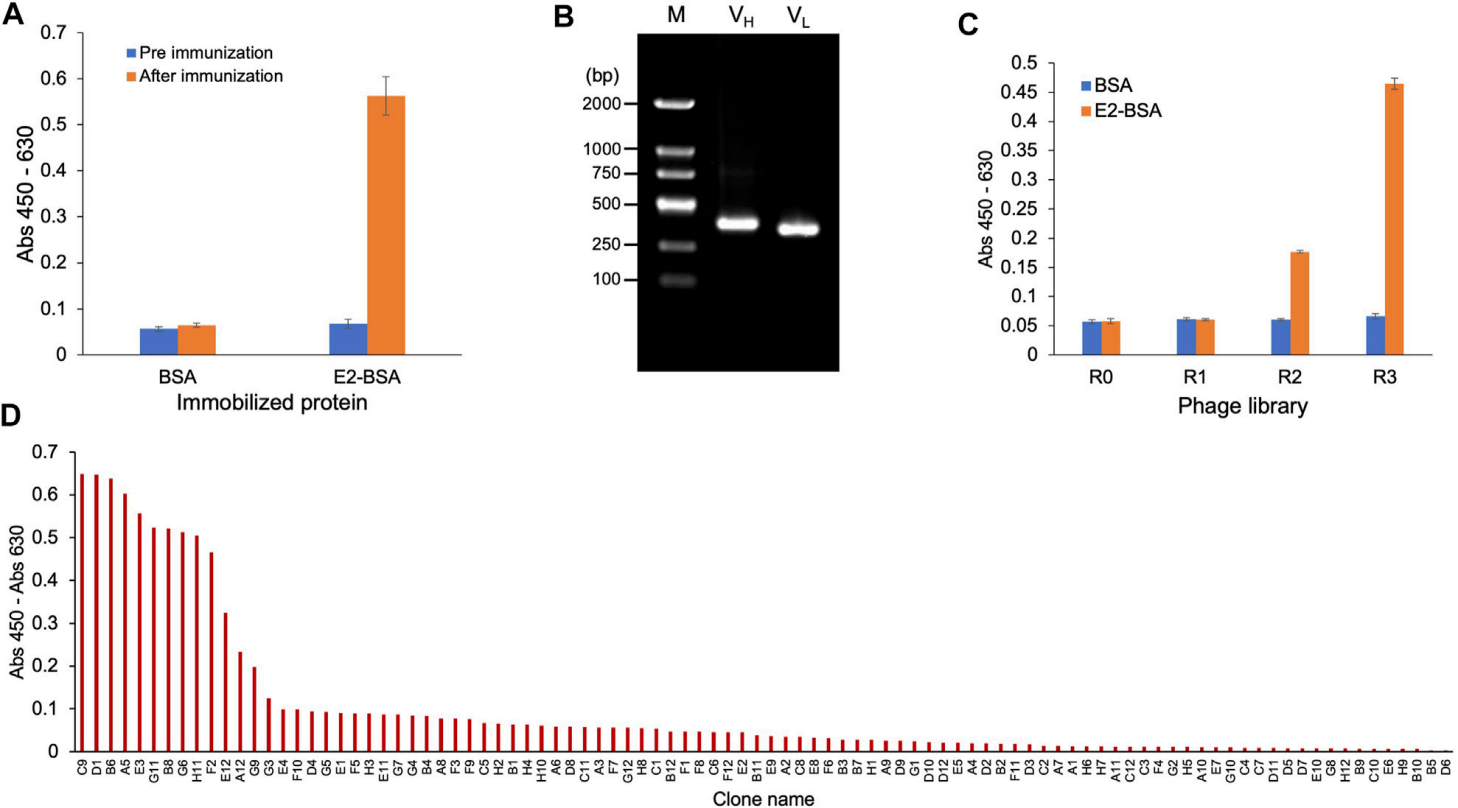
Cloning of anti-E2 antibody. **(A)** ELISA of anti-E2 antibody in the sera of mice before and after immunization; **(B)** Gene amplification product of V_H_ gene (400 bp) and V_L_ gene (slightly smaller than V_H_ gene); **(C)** ELISA of phage-displayed anti-E2 antibody; **(D)** ELISA of monoclonal antibodies screened from the phage displayed antibody library. E2: 17β-estradiol; M: DNA Marker (100–2000 bp); V_H_: variable regions of heavy chain; V_L_: variable regions of light chain; E2-BSA: conjugate of E2 and bovine serum albumin.

Venous blood was collected from the mice before and after three immunizations, and ELISA was performed. The results are shown in Figure 2A. After three immunization cycles, the absorbance of ELISA wells in which the serum from one mouse bound with E2-BSA was 0.58; this value was 9.7-fold higher than that of serum bound with BSA, which was only 0.06. Serum bound to BSA showed no signal difference before and after immunization, indicating that the signal difference observed in the serum bound to E2-BSA was due to the presence of specific antibodies against E2 after immunization, indicating that immunization was successful. Total RNA was extracted from the mouse spleens and used as a template to amplify the variable region genes of the antibodies. As shown in Figure 2B, the agarose gel electrophoresis results of PCR products with primers for the antibody V_H_ showed a DNA band of 400 bp, which was considered as the V_H_ gene. The amplified V_L_ gene with a slightly smaller size than that of V_H_ was also observed. The V_H_ and V_L_ genes were cloned into the phage display vector pDong1 and transfected into *E. coli* TG-1 to prepare a 2.1 × 10^6^ phage display antibody library. Three rounds of biopanning against E2-BSA were conducted, and ELISA was performed to identify the E2-BSA binding activity of the original phage display antibody library (R0) and three antibody libraries (R1, R2, and R3) obtained during biopanning. As shown in Figure 2C, as panning progressed, the binding activity of the phage library to E2-BSA gradually increased, whereas that of the negative control (BSA) did not increase, indicating that anti-E2 antibodies were enriched in the phage display antibody library. Phage R3 was used to infect *E. coli* TG-1, 96 clones were selected after culture, monoclonal phages were prepared, and a phage ELISA was performed. As shown in Figure 2D, ten clones showed strong binding activity to E2. DNA sequences of these antibody genes were same and considered from the same antibody, which was named as ESC9. No antibody with the same sequence was found in the antibody database, suggesting that ESC9 is a novel antibody.

### Construction of Fab Expression Vector

**FIGURE 3:**
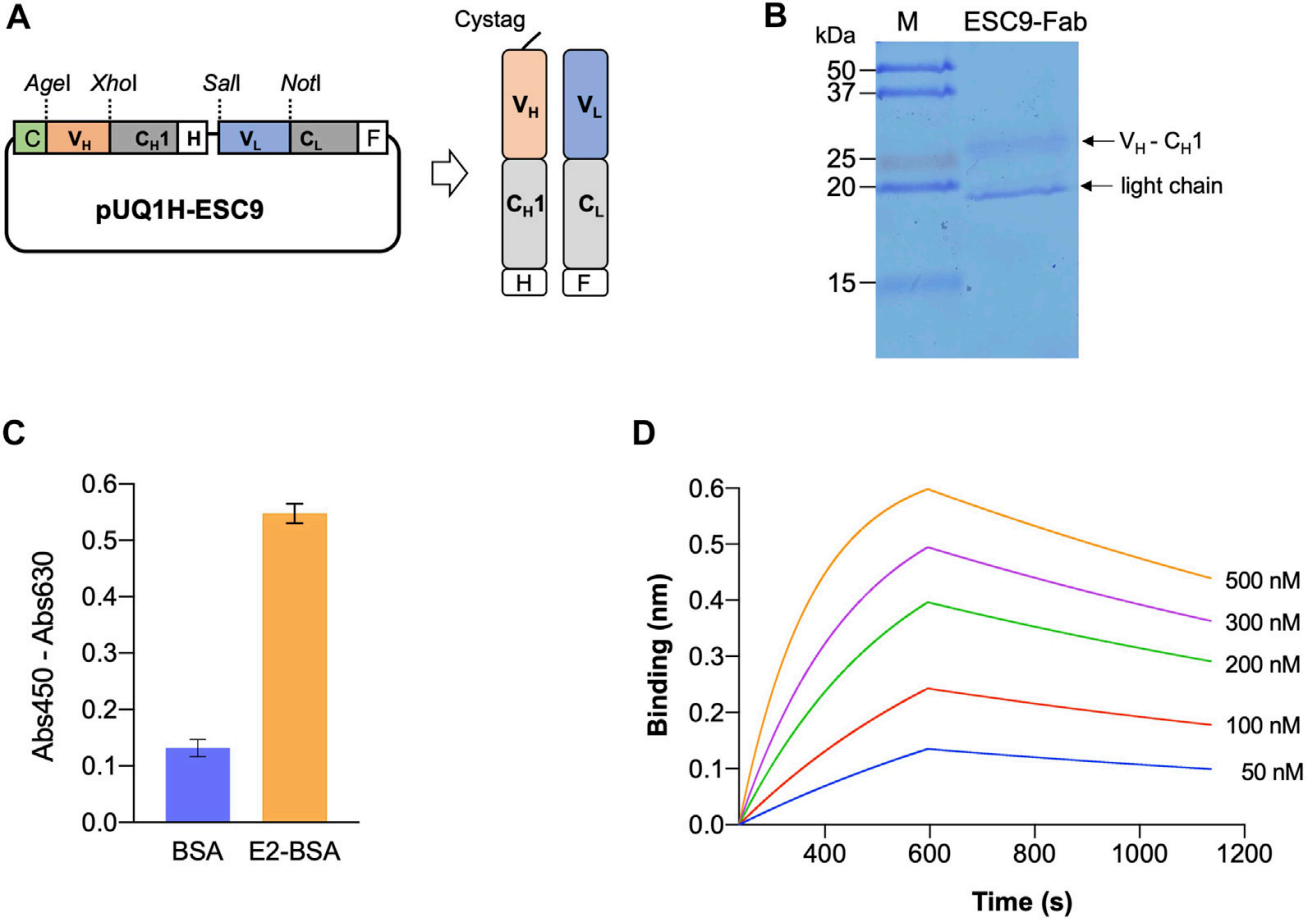
Expression of antigen-binding fragment of ESC9 monoclonal antibody. **(A)** Construct of Fab expression vector; **(B)** SDS-PAGE analysis of purified ESC9 fragment; **(C)** ELISA of antigen-binding activity of purified Fab; **(D)** Sensorgram of bio-layer interference analysis of ESC9 Fab binding to E2. C: Cys-tag1; H: His_6_-tag; F: Flag-tag; C_H_1: constant region 1 of human IgG1 heavy chain; C_L_: constant region of antibody light chain.

PCR was used to amplify the V_H-_C_H_1 and V_L_ fragments of the monoclonal antibody ESC9, and the amplicons were verified using agarose gel electrophoresis. As shown in Supplementary Figure S2A, for V_H_-C_H_1 and V_L_, DNA bands were observed at 800 and 400 bp, respectively. The amplicon size was consistent with the theoretical fragment size, and the target genes were eluted. The V_H-_C_H_1 and V_L_ fragments were then linked using an overlap PCR and verified using agarose gel electrophoresis, as shown in Supplementary Figure S2B. The target genes were purified and inserted into the target vector, and colony PCR was performed to screen for the expression vector pUQ1H-ESC9, as shown in Supplementary Figure S2C. The construction of Fab Expression Vector pUQ1H-ESC9 is illustrated in Figure 3A. A Cys-tag peptide (MAQIEVNCSNET) was attached to the N-terminal of the heavy chain of the vector to allow for efficient expression of the Fab of the antibody and modification of the fluorescent dye, and a 6×His-tag and a FLAG-tag (DYKDDDDK) at the C-terminal end of the antibody Fd fragment (V_H_-C_H_1) and light chain were included to purify the antibody Fab. The vector was successfully constructed, as confirmed using sequencing analysis.

### E2-Binding Affinity of ESC9-Fab

The Fab fragment of the ESC9 monoclonal antibody was purified using Ni-NTA Sefinose™ resin, and the purified Fab protein target bands was verified using SDS-PAGE. The results of Coomassie Brilliant Blue staining are shown in Figure 3B, where the Fd fragment of the heavy chain and light chain (V_L_-C_L_) of the Fab antibody were observed as two bands at 27 and 19 kDa, respectively. The size of Fd was similar to its theoretical molecular weight of 27.5 kDa, whereas its light chain was slightly smaller than the theoretical molecular weight of 24.4 kDa. This may be because we performed SDS-PAGE under non-denaturing conditions to prevent both proteins from stopping at the same position in the gel. The antigen-binding activity of ESC9-Fab was confirmed using ELISA. As shown in Figure 3C, purified Fab bound to E2-BSA with an absorbance of 0.57, whereas that against BSA was 0.12, indicating that purified Fab retained the antigen specificity of the original antibody. To further measure the kinetic parameters of ESC9 Fab binding to E2, E2-BSA-biotin was prepared, and its SA-binding activity was confirmed in a binding assay. As shown in Supplementary Figure S3, SA-HRP bound to E2-BSA-biotin but not to E2-BSA, indicating the successful preparation of E2-BSA-biotin. A sensorgram was obtained using BLI technology, as shown in Figure 3D, and the kinetic parameters are listed in Table 2. The association and dissociation rates were 1.32 ± 0.003 × 10^4^ M^−1^s^−1^ and 5.72 ± 0.07 × 10^−4^ s^−1^, respectively, and the *K*_D_ value was calculated as 43.3 ± 0.1 nM with a coefficient of determination (*R* ^*2*^) of 0.9908.

**TABLE 2:** Kinetic parameters of ESC9 Fab and Q-body for E2-binding.

	*K*_on_ (1/Ms) ×10^4^	*K*_dis_ (1/s) × 10^−4^	*K*_D_ (nM)	*R* ^2^
ESC9-Fab	1.32 ± 0.003	5.72 ± 0.07	43.3 ± 0.1	0.9908
ESC9 Q-body	1.26 ± 0.007	7.08 ± 0.06	56.2 ± 0.5	0.9783

### Cross-Reactivity of ESC9

The IC_50_ values of the competitive assays with E2, testosterone, dehydroepiandrosterone, pregnenolone acetate, cortisol, and diethylstilbestrol were calculated by fitting the results of a standard competitive ELISA. The IC_50_ values of the assay with E2 and testosterone were 796.5 and 16,547.0 ng/ml, respectively, whereas no competition was observed with the other compounds. Based on these results, the cross-reactivity of ESC9 against TS was 4.8%, whereas no cross-reactivity was observed for dehydroepiandrosterone, pregnenolone acetate, cortisol and diethylstilbestrol.

### Preparation of ESC9 Q-Body

**FIGURE 4:**
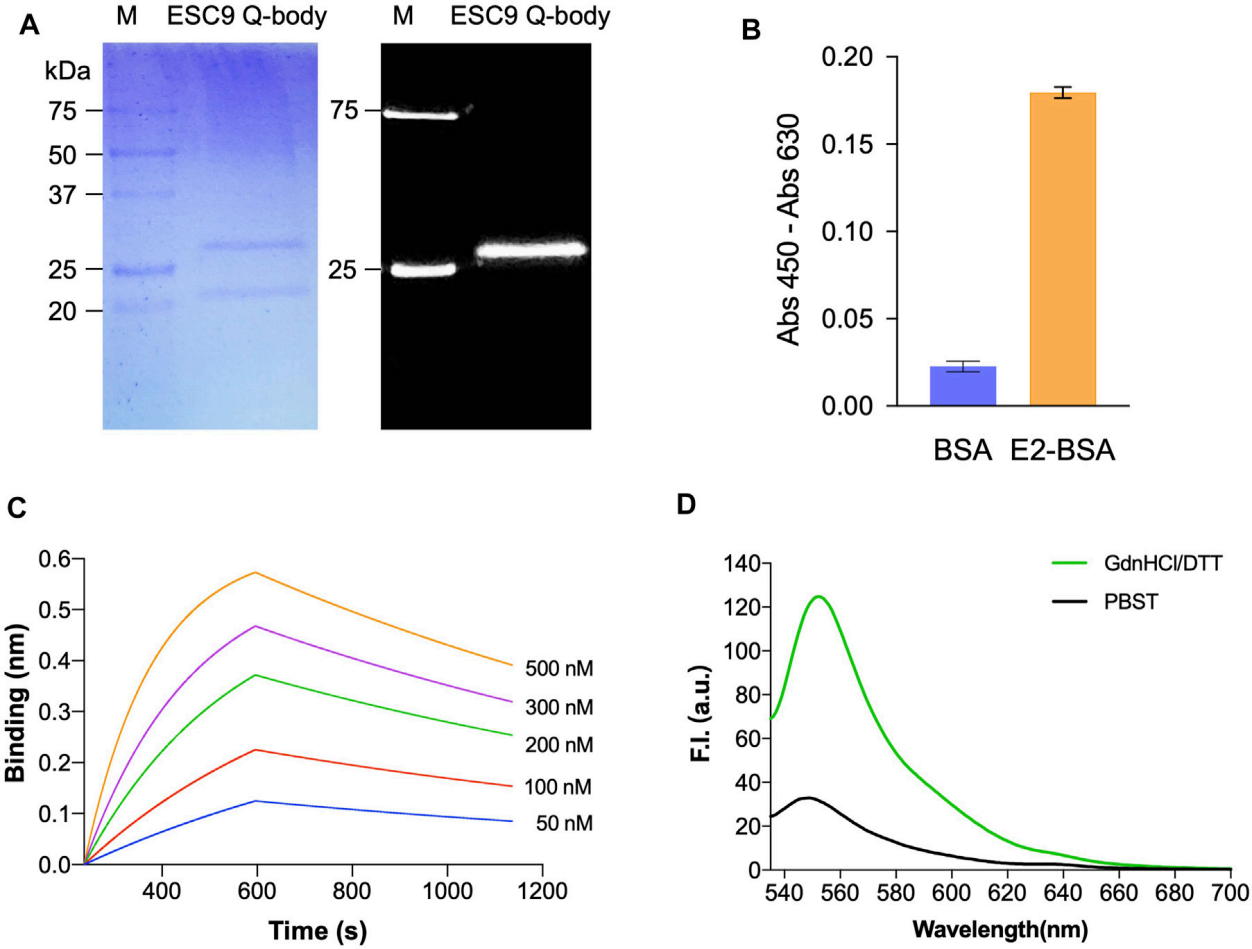
Analysis of Q-body sensor. **(A)** Coomassie Brilliant Blue staining and fluorescent image of separated Q-body in SDS-PAGE; **(B)** ELISA of antigen-binding activity of E2 Q-body; **(C)** Sensorgram of bio-layer interference analysis of ESC9 Q-body binding to E2; **(D)** Fluorescent spectra of Q-body in PBST and denaturing reagent. M: Precision Plus Protein™ Unstained Protein Standards with 1/50 of Precision Plus Protein™ Dual-color Protein Standards (Bio-Rad); PBST: PBS containing 0.5% Tween 20; GdnHCl/DTT: 7 M guanidine hydrochloride and 100 mM dithiothreitol in PBST.

ESC9 Fab was further labeled with ATTO520-C2-maleimide fluorescent dye and purified using G1 anti-DYKDDDDK beads. Fluorescent images and Coomassie Brilliant Blue staining of gels after SDS-PAGE were obtained to visualize the fluorescent modification of Q-bodies and their corresponding Fab target bands (Figure 4A). Two bands at 27 and 22 kDa were observed in the Coomassie Brilliant Blue-stained images and were considered to be the Fd and light chain, respectively. Only Fd was observed in the fluorescence image, suggesting that the labeling was successful. The antigen-binding activity of the ESC9 Q-body was assayed using ELISA with BSA as a negative control. The results are shown in Figure 4B, with an signal-to-noise value of 14.5, indicating that the fluorescence modification did not affect the antigen-binding activity of ESC9-Fab. Figure 4C shows the sensorgram of BLI analysis of ESC9 Q-body binding to E2, and the association rate, dissociation rate, and *K*_D_ values of the binding were calculated as 1.26 ± 0.007 × 10^4^ M^−1^s^−1^, 7.08 ± 0.06 × 10^−4^ s^−1^, and 56.2 ± 0.5 nM, with an *R* ^*2*^ of 0.9783, as shown in Table 2.

### Detection of E2 With ESC9 Q-Body

**FIGURE 5:**
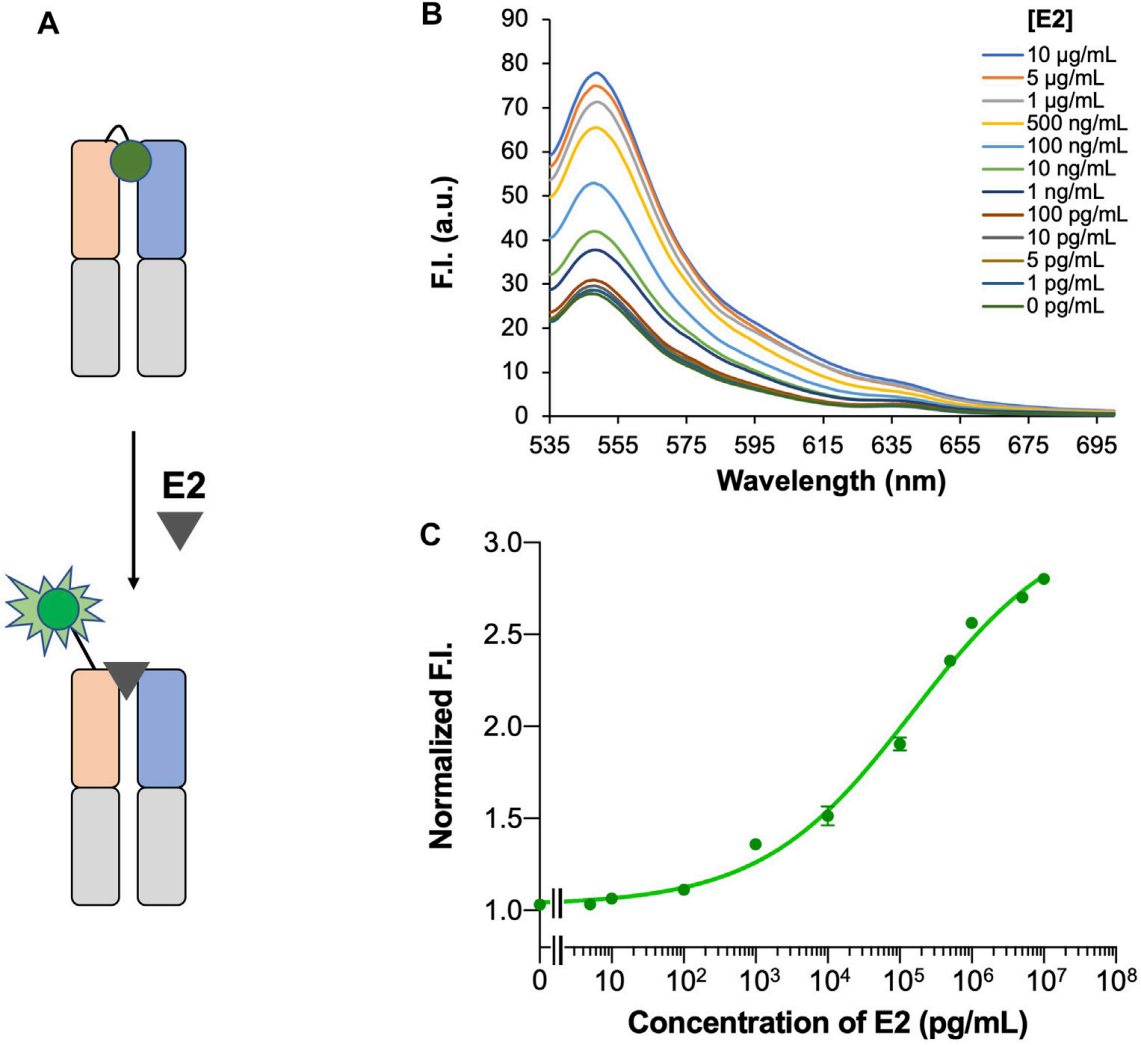
Detection of E2 with ESC9 Q-body. **(A)** Scheme for E2 detection with the ESC9 Q-body; **(B)** Fluorescent spectrum of the ESC9 Q-body for E2 detection; **(C)** Concentration-dependent relationship curve for the detection of E2 with ESC9 Q-body.

The quenching degree is an important parameter for evaluating the function of a Q-body (Ohashi et al., 2016). To evaluate the quenching degree, the fluorescence spectra of the ESC9 Q-body in PBST and denaturant GdnHCl/DTT were measured and compared. As shown in Figure 4D, the fluorescence intensity of the ESC9 Q-body at 545 nm was 3.8-fold higher than that of PBST, suggesting that the fluorescent dye in the ESC9 Q-body was quenched by the amino acids in the antibody. We then verified whether the fluorescence intensity increased upon E2 binding (Figure 5A). The fluorescence spectra of the ESC9 Q-body at different E2 concentrations are shown in Figure 5B. The maximum increase in fluorescence intensity was 2.8-fold at 10 μg/ml of E2; normalized fluorescence increase values at each concentration were plotted and fitted with a four-parameter to obtain an E2 concentration-dependent relationship standard curve, as shown in Figure 5C. Based on these results, the limit of detection of E2 and EC_50_ value were calculated as 3.9 pg/ml and 154.0 ng/ml, respectively.

### E2-Spiked Serum Test and Evaluation

Three E2 standards at concentrations of 100 pg/ml, 1 ng/ml, and 100 ng/ml were added to 25% serum to verify that the Q-body probe can be used to detect E2 concentrations in blood samples. The change in fluorescence of the Q-body probe in the serum was calculated after subtracting the serum background with no sample and was calculated with the concentration-dependent curve. As shown in Table 3, the spiked E2 concentrations of 0.1, 1.0, and 100.0 ng/mL were detected as 0.083, 1.27, and 105 ng/mL with recovery rates of 83.0%, 127 and 105%, respectively, indicate that the Q-body probe can be used to quantitatively determine E2 levels in serum.

**TABLE 3:** Recovery of spiked E2 in human serum.

Spiked conc. (ng/ml)	Detected conc. (ng/ml)	Recovery (%)	
		Rate	RSD
0.1	0.083	83	6.2
1.0	1.27	127	10.3
100	105	105	11.4

## Discussion

E2 is the most active natural estrogen among all endocrine disruptors and is often used as an important clinical indicator of gynecological diseases. Accurate quantification of serum estradiol levels is important for identifying physiological and pathological conditions in women. Moreover, E2 is commonly used as an endocrine-regulating drug in menopausal women (Adeel et al., 2017). When E2 accumulates in the human body through drinking water and food and exceeds the safety threshold, it destroys the body balance, damages human health, and endangers future generations (Singh et al., 2012; Zhang et al., 2013). Therefore, the research and development of E2 detection technology is of great significance.

In the present study, a novel monoclonal antibody against E2, ESC9, was developed by immunizing mice and constructing and biopanning a phage-display antibody library. The gene and allele symbols of the V_H_ of ESC9 were *IGHV1* and *IGHJ2*, respectively; those of the V_L_ region were *IGKV4* and *IGKJ2*. Using bio-layer interference, the affinity of ESC9 Fab was measured to be 43.3 nM, showing high affinity. Several anti-E2 antibodies have been reported previously. For example, Pajunen et al. reported three anti-estradiol-17β antibodies with Ka values of 2.2–13.4 nM (Pajunen et al., 1997). Kabayashi et al. reported an scFv antibody with an affinity of 860 nM developed by immunization with mouse and phage display technology, which was further improved to 26 nM using complementary-determining region shuffling technology (Kobayashi et al., 2008; Kobayashi et al., 2010). The affinity of our antibodies was equal to or slightly better than that of the aforementioned antibodies. In our further studies, we may perform artificial evolution of ESC9 using approaches such as molecular breeding (Oyama et al., 2013) or open-sandwich selection (Iwai et al., 2010) to further improve the affinity of the antibody and sensitivity of the assay. Although ESC9 does not cross-react with dehydroepiandrosterone, pregnenolone acetate, cortisol, and diethylstilbestrol, it does react with TS. We will artificially evolve antibodies to reduce cross-reactions and improve the antibody specificity, as reported previously (Liu et al., 2012).

By modifying the N-terminus of the ESC9 Fab fragment with a fluorescent dye, a Q-body for detecting E2 was prepared. The affinity of Q-body was measured to be 56.2 nM, which is slightly lower than that of ESC9 Fab, suggesting that fluorescent dye labeling did not greatly reduce antigen-binding activity. The Q-body assay detected E2 at concentrations as low as 3.9 pg/ml within 2 min. The Q-body assay is a homogeneous detection technology with excellent detection speed and sensitivity. Additionally, it is not necessary to remove other substances from the sample, and high-throughput automatic detection is easy to perform (Abe et al., 2011; Jeong and Ueda, 2014; Ueda and Dong, 2014). Although the detection sensitivity of our method is similar or slightly better than those of some previously reported assays mainly based on competitive immunoassays in which the detection time is generally long, and its detection range is relatively narrow. To date, only one homogeneous assay for detecting E2 has been reported (Chiu et al., 2011); however, the sensitivity was 1 nM (272 pg/ml). Compared with the traditional methods, the detection time and range were significantly improved in our assay. We also used the developed assay to successfully detect E2 in sera with excellent accuracy and recovery, indicating its potential for use in clinical applications.

## Data Availability

The original contributions presented in the study are included in the article/Supplementary Material, further inquiries can be directed to the corresponding authors.
