# Supplementary material for: Isolation of a Monoclonal Antibody and its Derived Immunosensor for Rapid and Sensitive Detection of 17β-Estradiol
Source: Front Bioeng Biotechnol. 2022 Mar 28;10:818983. doi: 10.3389/fbioe.2022.818983 (PMC8995505; doi:10.3389/fbioe.2022.818983)
Supplement: Supplementary file 1 [file DataSheet1.PDF]

Supplementary Information for

Isolation of a Monoclonal Antibody and its Derived Immunosensor for Rapid and Sensitive Detection of 17β-Estradiol

Jingru Liang[1†], Hang Dong[2†], Fei Xu[1], Baowei Li[1], Haimei Li[1], Limei Chen[1], Mei Li[1], Yingchu Liu[3], Guosheng Jiang[1,4*], Jinhua Dong[1,5*]

[1]*Key Laboratory for Biological Medicine in Shandong Universities, Weifang Key Laboratory for Antibodies Medicine, School of Life Science and Technology, Weifang Medical University, Weifang 261053, China*

[2]*School of Clinical Medicine, Peking University, Beijing 100191, China*

[3]*School of Clinical Medicine, Beijing University of Chinese Medicine, Beijing 100029, China*

[4]*College of Basic Medicine, Binzhou Medical University, Yantai 264003, China*

[5]*World Research Hub Initiative, Institute of Innovative Research, Tokyo Institute of Technology, Yokohama 226-8503, Japan*

[†] These authors have contributed equally to this work and share first authorship.

[*]Corresponding author.

Email: dongjh@wfmc.edu.cn (Jinhua Dong), jiangguosh@163.com (Guosheng Jiang)

Table of Contents

Table S1. Primers used to amplify mouse antibody V region S-2

Figure S1. Flow chat of construction of Fab-expressing vector S-3

Figure S2. Agarose gel electrophoresis analyses of purified antibody genes S-4

Figure S3. Analysis of biotinylated E2-BSA S-5

Table S1. The nucleic acid sequence of the primers used to amplify mouse antibody V region**.**

Name	sequence (5´-3´)
VH1	GGA ACC CTT TGG CCC AGC CGG CCA TGG CCS AGG TYC AGC TBC AGC AGT C
VH2	GGA ACC CTT TGG CCC AGC CGG CCA TGG CCC AGG TTC ACC TGC AGC ART C
VH3	GGA ACC CTT TGG CCC AGC CGG CCA TGG CCC AGG TRC AGC TGA AGG AGT C
VH4	GGA ACC CTT TGG CCC AGC CGG CCA TGG CCC AGG TCC AAC TVC AGC ARC C
VH5	GGA ACC CTT TGG CCC AGC CGG CCA TGG CCC AGA TCC AGT TGG TVC AGT C
VH6	GGA ACC CTT TGG CCC AGC CGG CCA TGG CCC AGG TGC AGC TGA AGS AST C
VH7	GGA ACC CTT TGG CCC AGC CGG CCA TGG CCG AGG TGC AGS KGG TGG AGT C
VH8	GGA ACC CTT TGG CCC AGC CGG CCA TGG CCG AAG TGA ARS TTG AGG AGT C
VH9	GGA ACC CTT TGG CCC AGC CGG CCA TGG CCG AKG TSV AGC TTC AGG AGT C
VH10	GGA ACC CTT TGG CCC AGC CGG CCA TGG CCG AGG TGA ASS TGG TGG AAT C
VH11	GGA ACC CTT TGG CCC AGC CGG CCA TGG CCG AGG TGA AGC TGR TGG ART C
VH12	GGA ACC CTT TGG CCC AGC CGG CCA TGG CCG ARG TGA AGC TGR TGG AGT C
VH13	GGA ACC CTT TGG CCC AGC CGG CCA TGG CCG AAG TGC AGC TGT TGG AGA C
VH14	GGA ACC CTT TGG CCC AGC CGG CCA TGG CCG ARG TGA AGC TTC TCS AGT C
VH15	GGA ACC CTT TGG CCC AGC CGG CCA TGG CCC ARG TTA CTC TGA AAG AGT
VK1	TAT TCG TCG ACG GAT ATT GTG ATG ACB CAG DC
VK2	TAT TCG TCG ACG GAT RTT KTG ATG ACC CAR AC
VK3	TAT TCG TCG ACG GAA AAT GTG CTC ACC CAG TC
VK4	TAT TCG TCG ACG GAY ATT GTG ATG ACA CAG TC
VK5	TAT TCG TCG ACG GAC ATC CAG ATG ACA CAG AC
VK6	TAT TCG TCG ACG GAY ATT GTG CTS ACY CAR TC
VK7	TAT TCG TCG ACG GAC ATC CAG ATG ACY CAR TC
VK8	TAT TCG TCG ACG CAA ATT GTT CTC ACC CAG TC
VL1	TAT TCG TCG ACG CAG GCT GTT GTG ACT CAG GAA TC

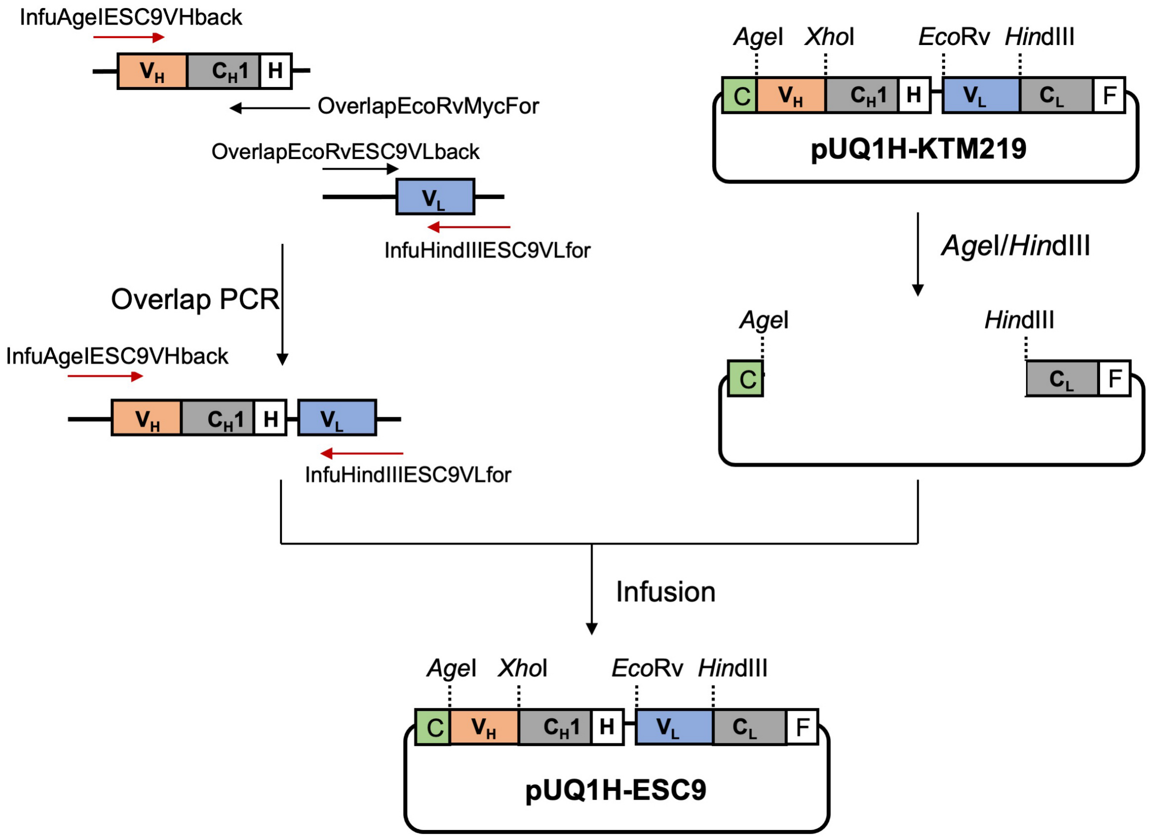

Figure S1. Flow chat of construction of Fab-expressing vector.

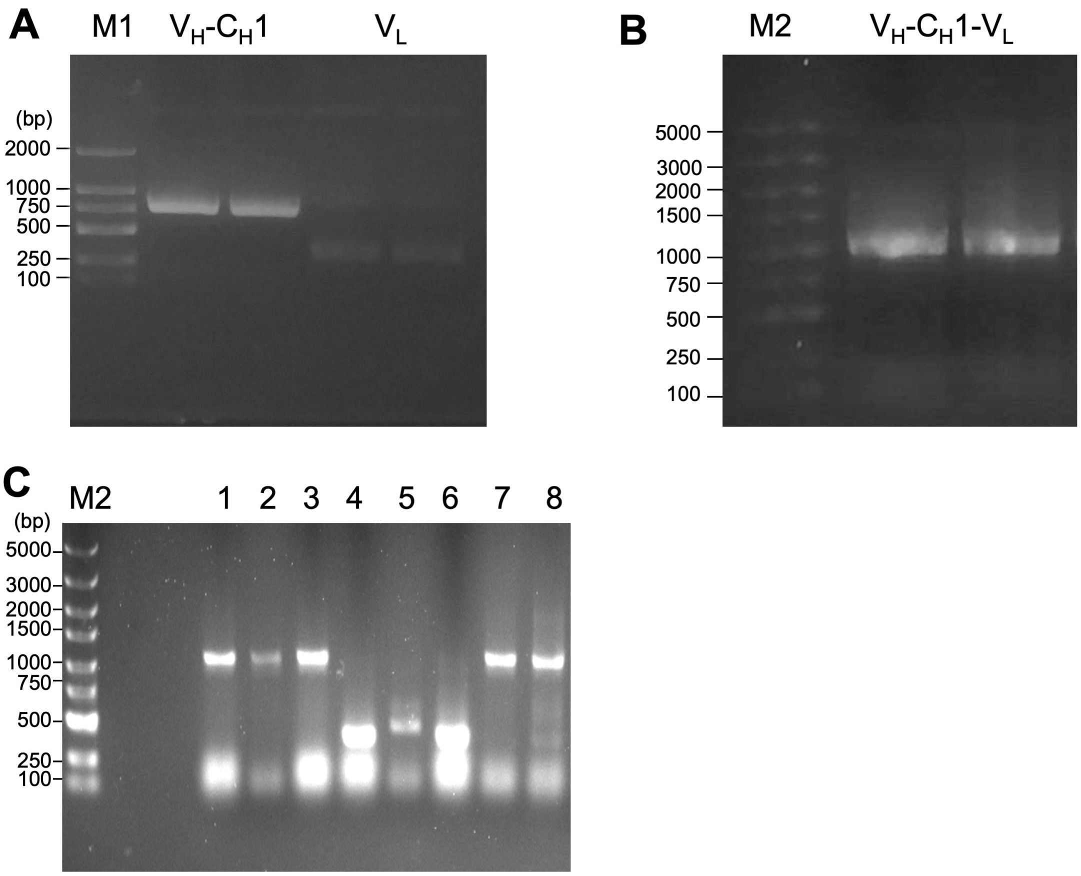

Figure S2. Agarose gel electrophoresis analyses of purified antibody genes. (**a**) Amplified V_H-C_H1 and V_L; (**b**) Amplified V_H-C_H1-V_L with an overlap PCR; (**c**)Colony PCR to screen positive clones. M1: DNA molecular weight standard Marker (100-2000bp); M2: DNA molecular weight standard Marker (100-5000bp) (Shanghai Sangon Biotechnology).

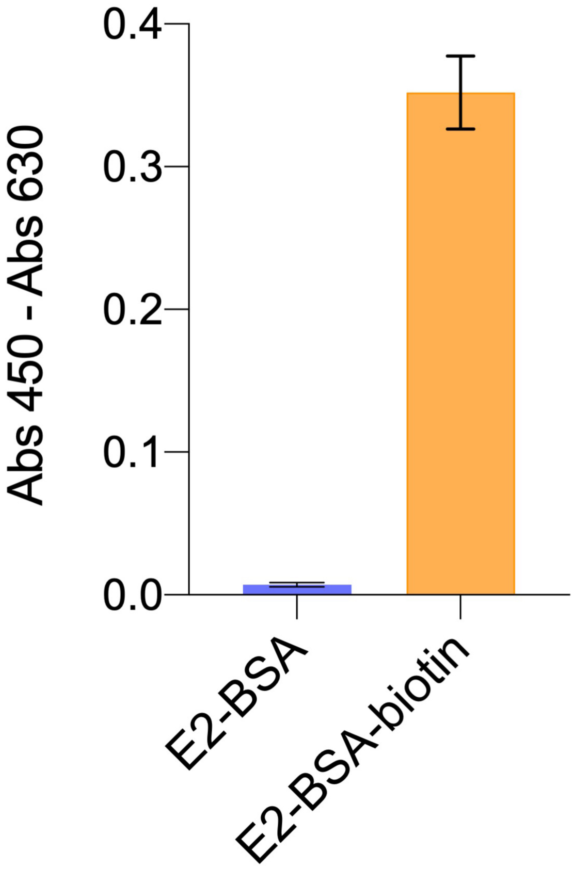

Figure S3. Analysis of biotinylated E2-BSA. E2-BSA and biotinylated E2-BSA were immobilized on a microplate at 10 μg/mL and HRP-conjugated streptavidin (Shanghai Sango Biotechnology) at 1μg/mL was used to detect biotinylation.
